# Research on an Infectious Disease Transmission by Flocking Birds

**DOI:** 10.1155/2013/196823

**Published:** 2013-06-24

**Authors:** Mingsheng Tang, Xinjun Mao, Zahia Guessoum

**Affiliations:** ^1^College of Computer, National University of Defense Technology, Changsha 410073, China; ^2^LIP 6, Université Pierre et Marie Curie, 75006 Paris, France

## Abstract

The swarm intelligence is becoming a hot topic. The flocking of birds is a natural phenomenon, which is formed and organized without central or external controls for some benefits (e.g., reduction of energy consummation). However, the flocking also has some negative effects on the human, as the infectious disease H7N9 will easily be transmited from the denser flocking birds to the human. Zombie-city model has been proposed to help analyzing and modeling the flocking birds and the artificial society. This paper focuses on the H7N9 virus transmission in the flocking birds and from the flocking birds to the human. And some interesting results have been shown: (1) only some simple rules could result in an emergence such as the flocking; (2) the minimum distance between birds could affect H7N9 virus transmission in the flocking birds and even affect the virus transmissions from the flocking birds to the human.

## 1. Introduction 

Swarm intelligence is becoming a hot research topic, which could be presented in natural, social, or artificial systems. It is often inspired by natural systems, especially biological systems. The flocking of birds is a classical phenomenon of swarm intelligence without central control [1]. How could birds fly in a flocking? Each bird only confirms the simple rules of alignment, cohesion, and separation, and then these birds could fly in a flocking. Why do birds fly in a flocking? Because it could reduce the power requirements of birds and could bring other benefits for these birds [2–4].

However, it also brings some problems, for example infectious diseases could spread quickly among the flocking birds. Moreover, these infectious diseases may be transmitted to the human such as the infectious disease H7N9 which is spreading in China [5]. This paper will study the wild flocking birds and its effect on the infectious disease spread from the viewpoint of the transmission of the infectious disease H7N9. In order to facilitate modeling the flocking and the spread of H7N9 between birds and human, this paper proposes a general model, zombie-city model, which contains five core concepts: agent, role, environment, social network, and rule. Based on this model, we could construct and analyze the artificial flocking birds and also could model the artificial society. We will study the spread of H7N9 in the flocking birds and H7N9 transmission from birds to the human.

This paper is organized as follows: Section 2 introduces the zombie-city model.  Section 3 models the flocking and artificial society with the zombie city and then presents the experiments results.  Section 4 summarizes the work.

## 2. Zombie-City Model

### 2.1. Meat-Model of Zombie-City Model

**Figure 1:**
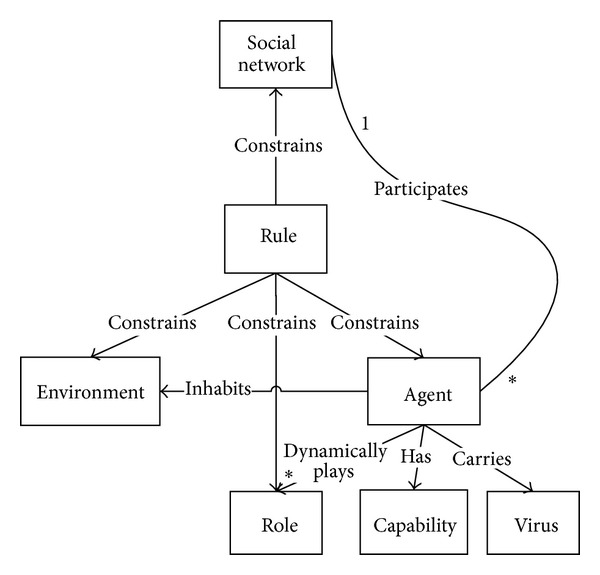
Metamodel of the zombie-city model.

In the zombie-city model [6], there are mainly five concepts, including agent, environment, social networks, roles, and rules. Rules could constrain agents, environments, and social network; that is, the agents, the environment, and the social network must conform to these rules. Besides, agents have their own capabilities (e.g., movement), and agents may be infected with viruses so that they will carry viruses to play the zombie roles.  Figure 1 presents the metamodel of the zombie-city model.

- * Agent*. It is a proactive, automatic, and self-adaptive entity. It could interact with other agents and has some capabilities such as interaction and sensing the social relationships with other agents. And agents could dynamically play different roles to adapt to the environmental, social, or their own changes.
- * Social Network*. It is made up of social relationships between agents, which construct the social structure. Meanwhile, it must conform to the rules such as scale-free network.
- * Environment*. It is the space where agents inhabit, which is made up of grids. The environment also must conform to the rules.
- * Rule*. It is constraining agents, social network, the environment and roles, and all of them do not act against the discipline or the norm.
- * Role*. It is the abstract of agents. In some emergent situations, we could define various special roles, for example, agents infected with viruses could be seen as the role of patient. Agents could dynamically play different roles according to various situations. Besides, roles should also be constrained by rules.
- * Capability*. It is the ability of agents such as movement and interaction. Agents could own some natural capabilities, including self-adaptive capability though dynamically playing different roles according to various situations.
- * Virus*. It is the entity that has not behavioral features and could be transmitted among agents by interactions or other ways. It could be considered as a property of agents to denote or indicate whether agents carry the virus. It is a very important concept for emergency management.

### 2.2. Formal Specification of the Zombie-City Model

A zombie-city model could be described by 5 tuples, as *Model* : : = 〈*AGENT*, *SN*, *EN*, *ROLE*, *RULE*〉, where

- *AGENT* = {*a*_1_, *a*_2_,…, *a*_*n*_}, for any *a*_*i*_(1 ≤ *i* ≤ *n*), where *a*_*i*_ is an agent and *AGENT* is a finite set of agents.
- *SN* = {*l*_*k*_ | 1 ≤ *k* ≤ (*n*−1)^2^}, where *l*_*k*_ is a link of the social network, *SN* is a set of links, and social network is constructed by links between these agents.
- *EN* = {*g*_1_, *g*_2_,…, *g*_*l*_}, where *g*_*i*_(1 ≤ *i* ≤ *l*) is a grid and *EN* is a finite set of grids.
- *ROLE* = {*b*_1_, *b*_2_,…, *b*_*m*_}, for any *b*_*i*_(1 ≤ *i* ≤ *m*), where *b*_*i*_ is a role and *ROLE* is a finite set of roles.
- *RULE* = {*r*_1_, *r*_2_,…, *r*_*k*_}, for any *r*_*i*_(1 ≤ *i* ≤ *k*), where *r*_*i*_ is a rule. *RULE* is a finite rules set of rules. This set could be divided into four subsets: *R*_*A*_, *R*_*S*_, *R*_*E*_, and *R*_*R*_. *R*_*A*_ is a finite rule set for agents, *R*_*S*_ is a finite rule set for the social network, *R*_*E*_ is a finite rule set for the environment, and *R*_*R*_ is a finite rule set for roles.

We use CID to express the set of all the identifications, and cid is a specific one, that is, cid ∈ CID.

- *Agent*. For any *a* ∈ *AGENT*, *a* : : = 〈*cid*, *AT*, *AC*, *N*_*l*_, *N*_*r*_〉, where
  (a) *cid* is the identification of the agent;
  (b) *AT* is the attributes of the agent;
  (c) *AC* is the actions of the agent that the agent could do;
  (d) *N*_*l*_ is a finite set of identifications of links that the agent is participating;
  (e) *N*_*r*_ means a set of identifications of roles that the agent is playing.
- *Role*. For any *r* ∈ *ROLE*, *r* : : = 〈*cid*, *AT*, *AC*〉, where
  (a) *cid* is the identification of the role;
  (b) *AT* is the attributes of the role;
  (c) *AC* is the actions of the role that the role could do.
- *Environment*. For any *e* ∈ *EN*,  *e* : : = 〈*cid*, *AT*, *AC*〉, where
  (a) *cid* is the identification of the environment;
  (b) *AT* is the attributes of the environment;
  (c) *AC* is the actions of the environment that the environment could do.
- *Social Network*. For any *l* ∈ *SN*, *l* : : = 〈*cid*, *AT*, (*a*_*i*_, *a*_*j*_)〉
  (a) *cid* is the identification of the link;
  (b) *AT* is the attributes of the link;
  (c) (*a*_*i*_, *a*_*j*_) ∈ 2^*AGENT*×*AGENT*^ denotes a link from agent *a*_*i*_ to agent *a*_*j*_. For an undirected graph, (*a*_*i*_, *a*_*j*_) = (*a*_*j*_, *a*_*i*_), and for directed graph, (*a*_*i*_, *a*_*j*_)≠(*a*_*j*_, *a*_*i*_).
- *Rule*. For any *u* ∈ *R*_*A*_ ⋃ *R*_*R*_ ⋃ *R*_*E*_ ⋃ *R*_*S*_, *u* : : = *Condition* | *Event* → (*AT* | *AC*), where
  (a) *Condition* is the internal state or attribute of the agent, role, environment, or link of the social network in the system;
  (b) *Event* is the set of events that happens in the system;
  (c) *AT* is the set of attributes of the agent, role, environment, or link of the social network in the system;
  (d) *AC* is the set of actions of the agent, role, environment, or other actions in the system.

### 2.3. Social Network

For an undirected graph, *a*≺_*t*_ *b* (*a*, *b* ∈ *AGENT*) means that agent *a* links with agent *b* at moment *t*, which is equal to *b*≺_*t*_ *a*. *LINK*(*a*, *t*) denotes the set of agents in which agent *a* connects with at moment *t*, that is, *LINK*(*a*, *t*) = {*b* | *a*≺_*t*_ *b*∧*b* ∈ *AGENT*}. 

For a directed graph, let *a*≺_*t*_ *b* (*a*, *b* ∈ *AGENT*) denote that agent *a* links to *b*, and agent *a* is the source of this link. *LINK* ^*S*^(*a*, *t*) denotes the set of agents that agent *a* connects, and agent *a* is the source of these links, that is, *LINK* ^*S*^(*a*, *t*) = {*b* | *a*≺_*t*_ *b*∧*b* ∈ *AGENT*}. Let *LINK* ^*T*^(*a*, *t*) indicate the set of agents that links to agent *a* at the moment *t*, and agent *a* is the target of these links, that is, *LINK* ^*T*^(*a*, *t*) = {*b* | *b*≺_*t*_ *a*∧*b* ∈ *AGENT*}. Therefore, the set of agents that agent *a* connects at moment *t* contains the agents that agent *a* links to and the agents that link to agent *a*, that is, *LINK*(*a*, *t*) = *LINK* ^*S*^(*a*, *t*)⋃*LINK* ^*T*^(*a*, *t*).

For describing the initialization process of a static social network and the growing process of a dynamic social network, we have defined two primitives: *Create*(), *Delete*(). *SN*(*t*) denotes the set of social links in an artificial society at the moment *t*.

(i) *Create*(*a*_*i*_, *a*_*j*_). For the undirected graph, *a*_*i*_ ⊀_*t*_ *a*_*j*_, that is, *a*_*j*_ ∉ *LINK*(*a*_*i*_, *t*) and *a*_*i*_ ∉ *LINK*(*a*_*j*_, *t*), *LINK*(*a*_*i*_, *t* + 1) = *LINK*(*a*_*i*_, *t*)⋃{*a*_*j*_} and *LINK*(*a*_*j*_, *t* + 1) = *LINK*(*a*_*j*_, *t*)⋃{*a*_*i*_}; for the directed graph, *a*_*i*_ ⊀_*t*_ *a*_*j*_, *LINK* ^*S*^(*a*_*i*_, *t* + 1) = *LINK* ^*S*^(*a*_*i*_, *t*)⋃{*a*_*j*_} and *LINK* ^*T*^(*a*_*i*_, *t* + 1) = *LINK* ^*T*^(*a*_*i*_, *t*), *LINK* ^*S*^(*a*_*j*_, *t* + 1) = *LINK* ^*S*^(*a*_*j*_, *t*), and *LINK* ^*T*^(*a*_*j*_, *t* + 1) = *LINK* ^*T*^(*a*_*j*_, *t*)⋃{*a*_*i*_}. For both two graphs, the link *l* = (*a*_*i*_, *a*_*j*_) and *SN*(*t* + 1) = *SN*(*t*)⋃{*l*}.
(ii) *Delete*(*a*_*i*_, *a*_*j*_). For the undirected graph, *a*_*j*_ ∈ *LINK*(*a*_*i*_, *t*) and *a*_*i*_ ∈ *LINK*(*a*_*j*_, *t*), *LINK*(*a*_*i*_, *t* + 1) = *LINK*(*a*_*i*_, *t*)∖{*a*_*j*_} and *LINK*(*a*_*j*_, *t* + 1) = *LINK*(*a*_*j*_, *t*)∖{*a*_*i*_}; for the directed graph, *a*_*j*_ ∈ *LINK* ^*S*^(*a*_*i*_, *t*) and *a*_*i*_ ∈ *LINK* ^*T*^(*a*_*j*_, *t*), *LINK* ^*S*^(*a*_*i*_, *t* + 1) = *LINK* ^*S*^(*a*_*i*_, *t*)∖{*a*_*j*_} and *LINK* ^*S*^(*a*_*j*_, *t* + 1) = *LINK* ^*S*^(*a*_*j*_, *t*), *LINK* ^*T*^(*a*_*i*_, *t* + 1) = *LINK* ^*T*^(*a*_*i*_, *t*) and *LINK* ^*T*^(*a*_*j*_, *t* + 1) = *LINK* ^*T*^(*a*_*j*_, *t*)∖{*a*_*i*_}. For both two graphs, the link *l* = (*a*_*i*_, *a*_*j*_) and *SN*(*t* + 1) = *SN*(*t*)∖{*l*}.

### 2.4. Self-Adaptive Mechanism of Agents

**Figure 2:**
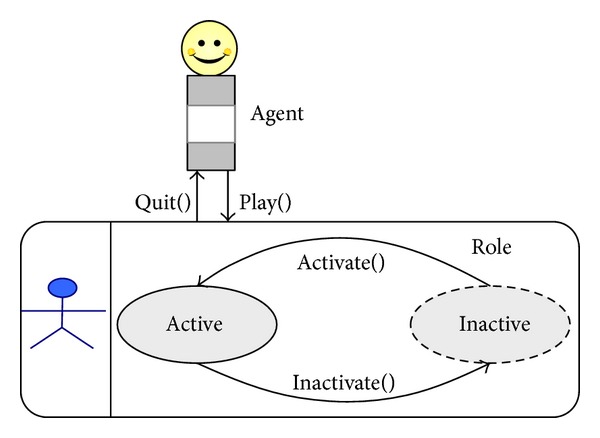
Mechanism of dynamically playing role.

Agents have the capabilities of self-adaption though dynamically playing roles.  Figure 2 presents the self-adaptive mechanism of dynamically playing roles. For example, if agent *a* is a student (i.e., agent *a* plays *student* role) and has been infected by an infectious disease, then agent *a* should adapt the change to transforming its role into *patient* role and go to a hospital. That means agent *a* will play the *patient* role, and the *student* role of agent *a* will be *inactive*. After agent *a* recovers, agent *a* will not play the *patient* role, that is, quit the *patient *role. Agent *a* will go back to play the *student* role, which means to *activate* the *student* role. The self-adaptive mechanism could be accurately described by formalization.

Let *a*∈_*t*_ *c* denote that agent *a* plays *c* role at time *t*. Use *ROLE*(*a*, *t*) to denote the set of roles that agent *a* plays at moment *t*, that is, *ROLE*(*a*, *t*) = {*c* | *a*∈_*t*_ *c*}. In order to describe the mechanism of dynamically playing roles, let *ROLE* ^*A*^(*a*, *t*) indicate the set of active roles that agent *a* plays at moment *t*, and let *ROLE* ^*I*^(*a*, *t*) denote the set of inactive roles that agent *a* plays at moment *t*. Therefore, the set of roles that agent *a* plays at moment *t* includes active roles and inactive roles, that is, *ROLE*(*a*, *t*) = *ROLE* ^*A*^(*a*, *t*) ⋃ *ROLE* ^*I*^(*a*, *t*). In order to describe the process of dynamically playing roles, we define four primitives, including *Play*(), *Quit*(), *Activate*(), and *Inactivate*().

 
*Play*(*c*). *c* ∉ *ROLE*(*a*, *t*), *ROLE* ^*A*^(*a*, *t* + 1) = *ROLE* ^*A*^(*a*, *t*)⋃{*c*}, and *ROLE* ^*I*^(*a*, *t* + 1) = *ROLE* ^*I*^(*a*, *t*). That means that the agent will play and join the role and the role is active.
 
*Quit*(*c*). *c* ∈ *ROLE* ^*A*^(*a*, *t*), *ROLE* ^*A*^(*a*, *t* + 1) = *ROLE* ^*A*^(*a*, *t*)∖{*c*}, and *ROLE* ^*I*^(*a*, *t* + 1) = *ROLE* ^*I*^(*a*, *t*)⋃{*c*}. That means that the agent will not play the role and the role will quit from the set of roles that the agent plays.
 
*Activate*(*c*). *c* ∈ *ROLE* ^*I*^(*a*, *t*), *ROLE* ^*I*^(*a*, *t* + 1) = *ROLE* ^*I*^(*a*, *t*)∖{*c*}, and *ROLE* ^*A*^(*a*, *t* + 1) = *ROLE* ^*A*^(*a*, *t*)⋃{*c*}. That means that the agent will make the role active and behaviors of the agent will be affected by the role.
 
*Inactivate*(*c*). *c* ∈ *ROLE* ^*A*^(*a*, *t*), *ROLE* ^*A*^(*a*, *t* + 1) = *ROLE* ^*A*^(*a*, *t*)∖{*c*}, and *ROLE* ^*I*^(*a*, *t* + 1) = *ROLE* ^*I*^(*a*, *t*)⋃{*c*}. That means that the agent will make the role inactive and behaviors of the agent will not be affected by the role.

**Figure 3:**
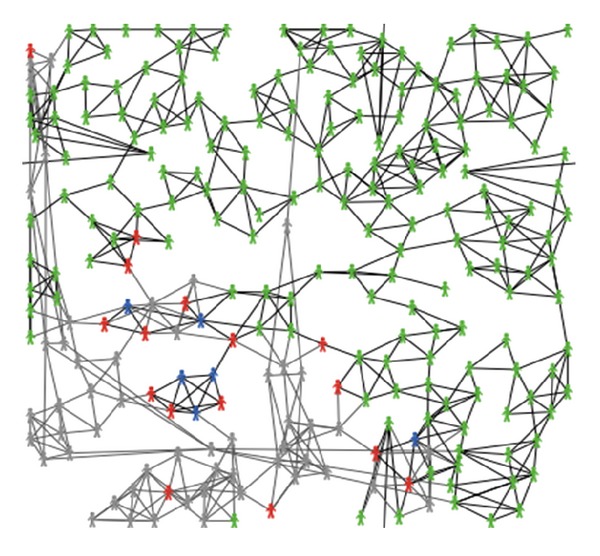
A snapshot of agents playing different roles (different colours) in the zombie-city model.

As presented in Figure 3, in zombie-city model, agents play different roles (indicated by different colors) and the same agent could dynamically play various roles for adapting to different situations.

## 3. H7N9 Spreading Based on the Flocking Birds

### 3.1. Modeling with the Zombie-City Model

The processes of the spread of H7N9 include the infectious disease transmitting among these flocking birds, and then the dense infected birds may transmit H7N9 to the human. In this case, there are mainly four roles: *susceptible_bird, infected_bird, susceptible_person*, and *infected_person*.

The phenomenon of flocking birds is based on some simple rules without external or central controls. This process is a self-organized process. These simple rules could be constructed as follows.

(1)* Rule_Cohesion*. If the bird is far away from its neighbors (flockmates) (*max_distance *is the threshold of the max distance from its neighbors), then this bird will turn towards its neighbors. The parameter *average_flockmates* means the average direction of the nearest neighbors, and *max_cohesion* means the adjusting degree of the direction of this bird. This rule could be described as follows:* Distance(self, nearest-neighbor) > max_distance → turn_towards(average_flockmates, max_cohesion).*

(2)* Rule_Separation*. If the bird is too close to the nearest neighbor (*min_separation* is the threshold of the min distance from the nearest neighbor), then turn away from this neighbor. This rule could be described as follows: *Distance(self, nearest-neighbor) < min_separation → turn_away(nearest_neighbor, max_separation).*

(3)* Rule_Alignment*. Keep the direction of the bird with the flockmates, and then turn towards its neighbors. The parameter *max_ alignment* means the adjusting degree of the direction of this bird. This rule could be described as follows: *Distance(self, nearest-neighbor) < max_distance ∣∣ Distance(self, nearest-neighbor) > min_separation → turn_towards(average_flockmates, max_alignment).*

**Figure 4:**
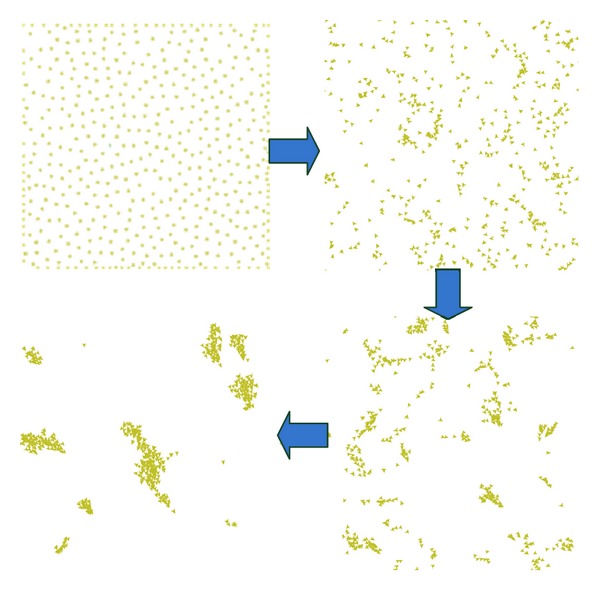
The processes of the flocking.

As shown in Figure 4, based on these three rules, the flocking will appear.

As we know, H7N9 is highly pathogenic for birds; that is, the infectious disease H7N9 could easily infect the birds. If some birds interact with the bird infected with H7N9 and the distance between these birds is much closed, then all of these birds will be infected with H7N9. So the rule of the disease spreading between birds could be described as follows.

(4)* Rule_Birds_Infected*. If the infected bird is closed with other birds and the distance from the infected bird (*Infected_bird*) to other birds without infection (*Susceptible_bird*) is less than a threshold (*min_distance*), then all these birds without infection will be infected with H7N9, that is, ∀*a*∈_*t*_ *Infected_bird *∀*b*∈_*t*_ *Susceptible_bird Distance(a,b) < min_distance → b.Quit(Susceptible_bird) *∧* b.Play(Infected_bird).*

As the recent research shows, the infectious disease H7N9 has almost spreaded from the wild birds to the human, and there is no evidence of ongoing human-to-human infection [5, 7, 8]. It could be assumed that human-to-human transmission is only happening between intimate people and the probability of infection is very small. For people, the number of strong social relationships of a person is also very small, and in this case we assume the number to be 1. Usually, these intimate people live nearby. So the rule of this social network of artificial society could be described as follows.

(5)* Rule_Social_Network*. This social network is an undirected graph, and an agent connects with the nearest agent which has not connected with it, that is,* Average_degree < 1 → *(∀*a*, *c*∈_*t*_ *(Infected_person *∨* Susceptible_person) *∃*b*∈_*t*_ *(Infected_person *∨* Susceptible_person) *(*a*, *c*)∉* LINK* ^*t*^ *(a,t) *∧* (a,b) *∉* LINK(a,t) *∧* Distance(a,b) < Distance(a,c) → Create(a,b)).*

Compared with the flocking birds, the positions of people in the environment could be seen as static. The wild flocking birds with H7N9 viruses will infect the human, but there are some conditions: in the unit grid of a person living, there are a number of infected birds (the number is *threshold_infected_human*).

(6)* Rule_Birds_Human_Infected*. If the agent without infection is habiting in a gird and in the gird there are enough infected birds (*threshold_infected_human*), then the agent will be infected, that is, ∀*a*∈_*t*_ *Susceptible_person *∃*b*∈* Environment (Habit(a,b) *∧* Count(Infected_birds,b) ≥ threshold_infected_human) → a.Quit(Susceptible_person) *∧* a.Play(Infected_person).*

Although there is no evidence of ongoing human-to-human transmission, we could not exclude the small probability of human-to-human transmission through the intimate social network.

(7)* Rule_Human_to_Human*. If an infected agent *a* has connected with a susceptible agent *b* and the randomly produced number is less than the threshold (*infected_chance*), then this agent *b* will be infected with H7N9, that is, ∀*a*∈_*t*_ *Infected_person *∀*b*∈_*t*_ *Susceptible_person ((a,b) *∈* LINK(a,t) *∧* Random() < infected_chance) → b.Quit(Susceptible_person) *∧* b.Play(Infected_person).*

### 3.2. Experiments

Based on these defined rules, we could do simulations and see the situations of the spread of this infectious disease H7N9.

- *Agents*. The population of agents is 750. The number of the humans is 250, and the number of birds is 500.
- *Environments*. 32 × 32 grids.
- *Role*. *Susceptible_bird (yellow color), susceptible_person (green color)*, *infected_bird (red color)*, and *infected_person (red color)*.

**Figure 5:**
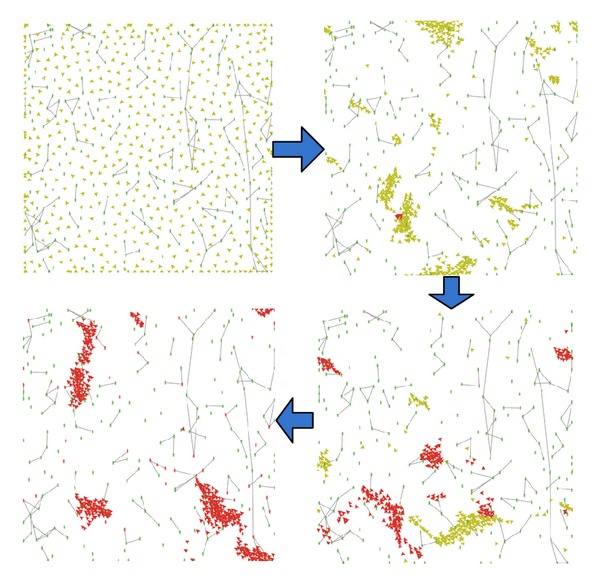
The processes of H7N9 transmission.

We define the parameters as follows: *min_separation = max_distance = 0.5, max_cohesion = 3, max_alignment = 5, max_separation = 1.5, infected_chance = 0.1, threshold_infected_human = 5*, and the average of the social network of the human is 1.  Figure 5 shows the processes of H7N9 transmission between the flocking birds and the human.

**Figure 6:**
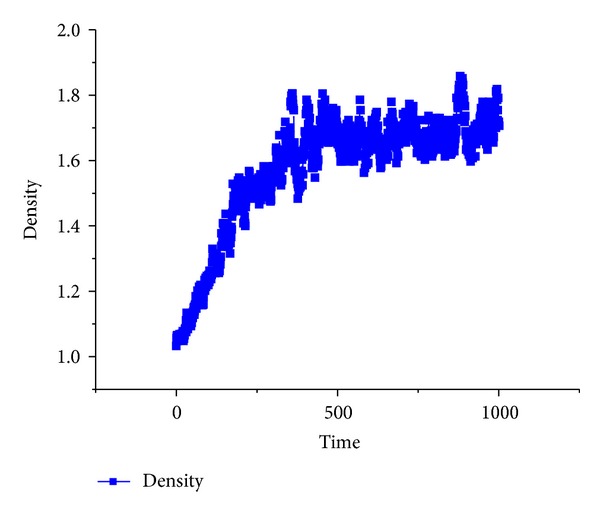
Density of the flocking birds.

As shown in Figure 6, the density of the flocking birds firstly linearly increases, and then it fluctuates around 1.7. This density is related to the parameter *min_separation*. 

**Figure 7:**
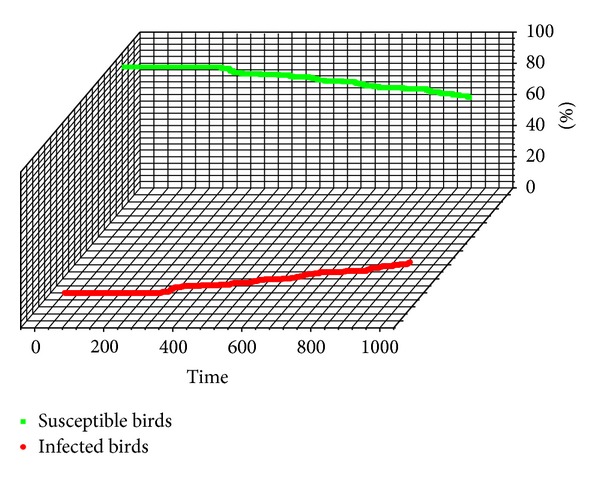
The epidemic status of the flocking birds.

Figure 7 shows the status of the H7N9 spreading between the flocking birds. At the ticks (200), a bird was infected with H7N9, and then the viruses transmitted among these birds quickly. At the ticks (400), all of these birds were infected with H7N9.

**Figure 8:**
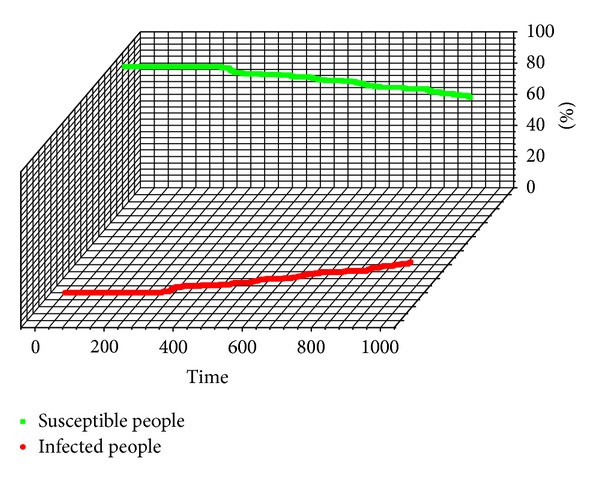
The epidemic status of the human.

After about 80 ticks of the first bird infection with H7N9, the first person was infected with H7N9 by the dense flocking birds. The number of the infected people is increasing slowly. The birds-to-human transmission is the main way for H7N9 transmitting from birds to the human, and the epidemic status of the human could be shown in Figure 8.

**Figure 9:**
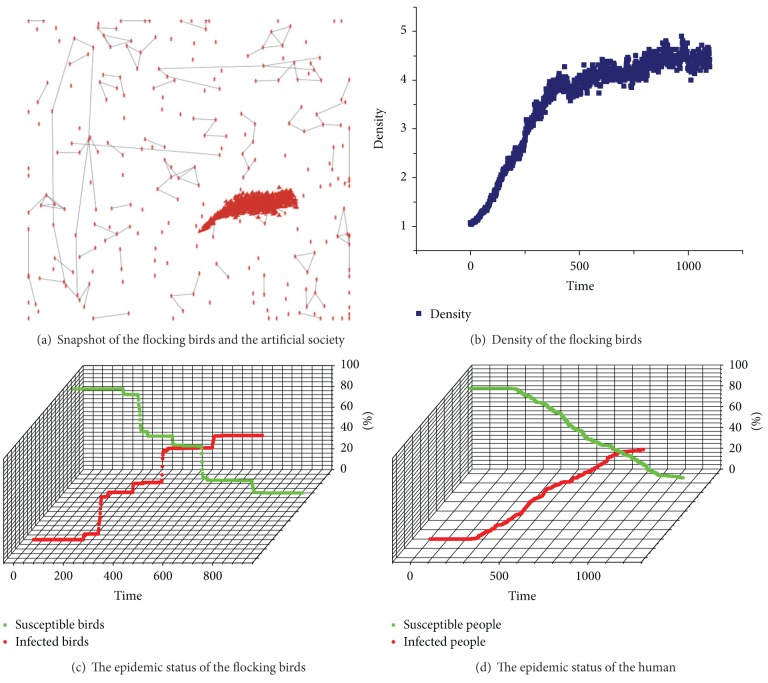
The simulation results with the parameter *min_separation* = 0.25.

How does the parameter *min_separation* affect the flocking birds and even the transmission of H7N9? We could adjust the parameter *min_separation *to 0.25. It means that the flocking birds will fly closed and the density of birds will increase. As shown in Figure 9, (a) presents the screenshot of the simulation; (b) shows the density of the flocking birds, and the density linearly increased and fluctuated around 4.5; (c) and (d) show the epidemic statuses of the flocking birds and the human, respectively.

As shown in Figure 9, we could clearly see that after the first bird was infected with H7N9 at the 200 ticks and at the 755 ticks, all of the birds were infected with H7N9. Because a small number of birds were fling separatly and very closly, the birds in some small flocking were not infected with H7N9 until these birds met with the bigger flocking. Moreover, in human, more people were infected with H7N9 for the bigger density of birds or the denser flocking birds. During a same time, the number of infected people with *min_separation* = 0.25 is four times that of the number of infected people with *min_separation* = 0.5. Above all, we could know that the density of the flocking birds is related to the H7N9 epidemic in the human. 

## 4. Conclusion

The phenomenon of the flocking is a natural beautiful scene. This phenomenon is organized by the birds without external and central controls. However, the flocking also has some problems, for example, the infectious disease H7N9 transmission. In order to aid analysis and modeling of the flocking and the disease transmission, this paper proposes a new artificial society model, zombie-city model, which includes five key concepts: agent, role, environment, social network, and rule. Based on this model, three simple rules of the flocking and other rules have been described. Then, this paper has studied the infectious disease H7N9 transmission among the flocking birds and the human. The simulation results show that a larger density of the flocking could accelerate the spread of the infectious disease H7N9 transmission from the flocking to the human and could slow down the spread of the H7N9 in the flocking birds.

The next works will include the following. (1) We will continue to perfect this zombie-city model, especially the description of an emergence. (2) We will continue with the flocking algorithm based on the zombie-city model and its effects on the human.
